# Can we predict favourable quality of life after surgically treated vertebral osteomyelitis? Analysis of a prospective study

**DOI:** 10.1007/s00402-022-04431-3

**Published:** 2022-03-31

**Authors:** A. Yagdiran, C. Otto-Lambertz, B. Sondermann, A. Ernst, D. Jochimsen, R. Sobottke, J. Siewe, P. Eysel, N. Jung

**Affiliations:** 1 Department of Orthopedic and Trauma Surgery, Faculty of Medicine and University Hospital Cologne, University of Cologne, Kerpener Str. 62, 50937 Cologne, Germany; 2 Faculty of Medicine and University Hospital Cologne Institute of Medical Statistics and Computational Biology, University of Cologne, Cologne, Germany; 3 Division of Infectious Diseases, Department I of Internal Medicine, Faculty of Medicine and University Hospital Cologne, University of Cologne, Cologne, Germany; 4 Department for Spine Surgery, Neurosurgery and Orthopedics, Rhein-Maas Klinikum GmbH, Mauerfeldchen 25, 52146 Würselen, Germany; 5 Department for Spine Surgery, Klinikum Leverkusen gGmbH, Am Gesundheitspark 11, 51375 Leverkusen, Germany

**Keywords:** Spondylodiscitis, Oswestry Disability Index (ODI), Quality of life (QoL), Patient-related outcome measures (PROMs), Predicting factors

## Abstract

**Purpose:**

Vertebral osteomyelitis (VO) is a severe clinical entity associated with significant morbidity and mortality. Several studies have showed that successful treatment of VO patients leads to significantly improved quality of life (QoL). Nevertheless, QoL levels of these patients remained below those of the general population. There are rarely studies focusing on predicting factors for favourable QoL after surgically treated VO. The aim of this study was to identify factors influencing positively the QoL of patients undergoing surgery for VO.

**Methods:**

We conducted a prospective monocentric study including surgically treated VO patients from 2008 to 2016. Data were collected before (T0) and 1 year (T1) after surgery. Primary outcome was favourable QoL defined as back pain with disability restricting normal life activity with a cutoff value ≥ 12 on Oswestry Disability Index (ODI).

**Ethics:**

Ethical approval was given by the Faculty of Medicine at the University of Cologne (09-182).

**Results:**

A total of 119 patients surviving 1 year after surgically treated VO were analysed. Favourable QoL was achieved in 35/119 patients. On multivariate analysis, younger age (hazard ratio = HR: 0.95; 95% CI 0.91–0.99; *p* = 0.022), lower albumin (HR: 0.9; 0.83–0.98; *p* = 0.019) an ASA score ≤ 2 (HR:4.24; 95%CI 1.42–12.68; *p* = 0.010), and a lower preoperative leg pain on the VAS (HR: 0.86; 95% CI 0.76–0.97; *p* = 0.018) were identified as independent risk factors for favourable QoL. Interestingly, the absence of neurological deficits was not predictive for a favourable outcome by means of QoL.

**Conclusion:**

One-third of surgically treated VO patients (29%) in our cohort achieved favourable QoL by means of ODI. Our findings can facilitate an estimation of the prognosis when informing the patient before surgery, and underscore that spine disability questionnaires, such as ODI, measuring QoL, are mandatory to evaluate comprehensively the outcome of this entity.

## Introduction

Vertebral osteomyelitis (VO) is a severe clinical entity associated with significant morbidity and mortality [1]. Several studies have shown that successful treatment of VO patients leads to significantly improved quality of life (QoL) and a significant reduction of back pain [2]. Nevertheless, QoL levels of these patients remained below those of the general population and seem to be comparable to those of chronic back pain patients [1, 2]. Various scores can be used to measure QoL, in which the Oswestry Disability Score (ODI) represents the internationally most recognized questionnaire for back pain [3]. Tonosu et al. showed that an ODI value between 0 and 12 is associated with back pain without disabilities, which allows a normal everyday life. However, a cutoff value above 12 points represents a condition with back pain and disabilities leading to a restriction in daily life [4].

There are only few studies focusing on a favourable outcome or on predicting factors for a successful surgical treatment of VO, in particular addressing means of QoL. Available data are mainly based on retrospective analyses and inhomogenous populations where the assessment of risk factors for sequels after surgery remains unclear.

In this context, the goal of this study was to assess factors influencing favourable QoL of surgically treated VO patients by means of ODI, which were recorded in a prospective study.

## Materials and methods

### Patient selection

From 2008 until 2016, patients with diagnosed VO at the Department for Orthopaedics and Trauma at a tertiary referral hospital were enrolled into the European “Spine Tango” register. Study inclusion criteria were the presence of characteristic back and/or leg pain plus characteristic magnetic resonance imaging (MRI) or an abscess or vertebral body destruction detected by computed tomography (CT). All cases were discussed interdisciplinarily between an infectious diseases specialist and an orthopaedic surgeon to confirm the diagnosis of VO. Cases that were not clearly diagnosed as VO by our strict criteria, but showed other underlying diseases (e.g. tumour or osteoporotic fractures) were excluded from the study. Adult patients (≥ 18 years of age) with indications for surgery were considered for analysis.

### Data collection

The following data were prospectively collected after enrolment in the European “Spine Tango” register: age, sex, length of hospital stay, affected spine segment and American Society of Anesthesiologists (ASA) score. The ASA Physical Status Classification System was developed in 1941 to classify patient comorbidity and is widely used by clinicians. ASA Class I is defined as a normal healthy patient, and Class V as a moribund patient not expected to survive without surgery. To assess QoL, the ODI was used. The ODI is a self- administered questionnaire and consists of tenitems. The first section rates the intensity of pain, whereas the rest describes its disabling effect on typical daily activities. The sum of the ten scores results in a score from 0 to 100. A lower average score demonstrates a higher QoL. The ODI score was collected preoperatively (T0) as well as after 1 year (T1). To measure the intensity of the pre-operative pain, visual analogue scale (VAS) from the Core Outcomes Measures Index (COMI) was used. The COMI consists of six items including two visual analogue scales (VAS), measuring back and leg pain that result in a score from 0 to 10.

In addition, the following variables were recorded for all VO patients undergoing surgery: bacteraemia, causative pathogens, body mass index (BMI), relevant comorbidities (diabetes, oncologic disease, immunosuppression, chronic obstructive pulmonary disease = COPD, inflammatory bowel disease = IBD, rheumatic disease, cardiac insufficiency, renal insufficiency, endocarditis) and conditions (alcohol and drug abuse), laboratory, radiological data, presence of psoas abscess or empyema and pre-operative neurological deficits based on the Frankel scale. The Frankel scale classifies the extent of the neurological/functional deficit into five grades. Grade A shows no motor or sensory function below the level of lesion, whereas Grade E is defined as a normal motor and/or sensory function.

### Definition of end points

Primary end point was favourable QoL defined as an ODI score < 12 points 1 year (T1) after surgery according to the reported cutoff values for back pain without disabilities allowing a normal everyday life [4].

### Surgical procedures

VO patients with bony destruction of the endplates, suspected instability and intraspinal empyema were treated surgically. In patients with progressive neurological failures with or without impending sepsis or haemodynamic instability, surgical intervention was performed immediately. Further indications were pain caused by spinal instability, progressive deformity, and/or intraspinal empyema, or failure to respond to conservative antimicrobial therapy. The surgical procedure was chosen according to vertebral destruction. If only an abscess or even an empyema was present, intraspinal debridement was performed. In cases of bony destruction and/or instability, additional intercorporal fusion was carried out. If a reconstruction of the ventral column was indicated but not possible from the dorsal approach due to the extent of the bony destruction, a two-staged treatment was chosen: debridement and instrumentation in the first surgery and corporectomy/spondylodesis with implantation of iliac crest grafts, intervertebral cages or vertebral body replacement in the second surgery (ventral approach). In the lumbar spine, fusion was performed in one stage in a posterior lumbar interbody fusion (PLIF) or in two stages in an anterior lumbar interbody fusion (ALIF) technique.

### Antibiotic treatment

Whenever possible, antimicrobial therapy of VO was directed against the identified specimen. In culture-negative cases empirical therapy was administered with our local standard regimen (ceftriaxone and additional administration of flucloxacillin) as MRSA rates are low in Germany. In general, postoperative antibiotic treatment was administered intravenously for 14 days, followed by a highly bioavailable oral antimicrobial therapy oralization with a duration of 6–12 weeks in total depending on the severity of disease deferred response or risk factors (e.g. foreign material-associated infection). A longer intravenously therapy was applied when indicated (e.g. concomitant endocarditis; *Staphylococcus aureus* bacteraemia; disseminated infectious complications). Therapy for specific microorganisms was applied according to the guidelines (e.g. *Mycobacterium *spp*., Candida *spp*., Brucella *spp*.*) [5–7].

### Statistical analysis

All statistical analyses were performed using IBM SPSS Statistics Version 26. Categorial variables were compared using *χ*^2^ or Fisher`s exact test, as appropriate. Ordinal data are compared by median test for independent samples. To identify independent factors for a favourable outcome (ODI < 12 points), all variables found to be clinically relevant or univariately significant were included in a multivariable logistic regression model. Variable selection (forward and backward) was then applied and the best model according to the Akaike information criterion (AIC) was selected. All statistical tests were two- tailed and *p* ≤ *0.05* was considered to indicate statistical significance. Statistical analyses were performed in an explorative descriptive sense.

## Results

**Fig. 1:**
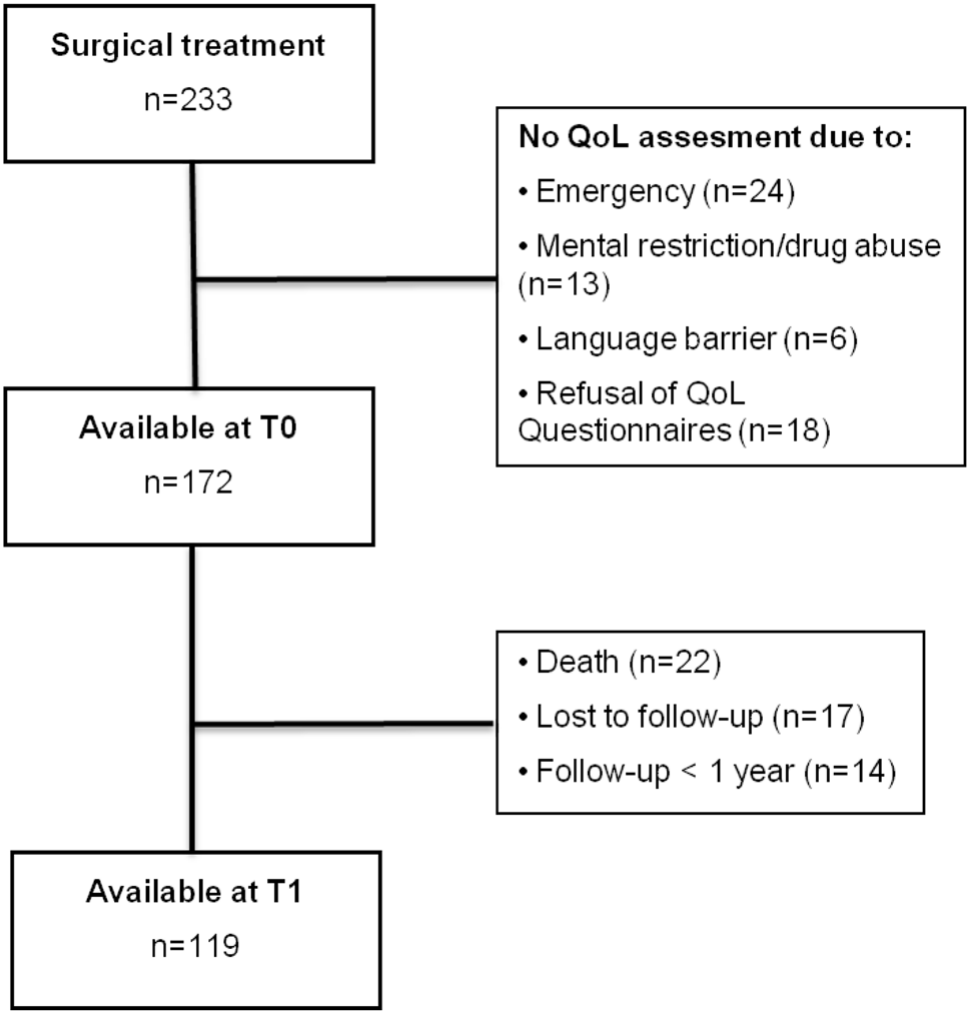
Flowchart for patient selection

From 2008 until 2016, 233 inpatients with confirmed VO were surgically treated in our department. A pre-operative ODI assessment (T0) was possible in 172 patients. After 1 year,119 patients were available for evaluation at T1 (Fig. 1).

Mean ODI values improved significantly from 75.4 (SD ± 18.5) at T0 to 29.1 (SD ± 22.1) at T1 (*p* < 0.001). In five patients (4%), a pure decompression was sufficient. In 51 patients (43%), a one-stage procedure was possible including 26 cases (22%) of PLIF. In 68 patients (57%), a two-stage procedure for ventral stabilization was necessary including 43 (36%) ALIFs. In 61 cases (51%), corporectomy was performed. The baseline characteristics of patients with favourable and unfavourable QoL are shown in Table 1.

**Table 1:** Patient characteristics and clinical data at baseline of 119 patients with surgically treated vertebral osteomyelitis (VO) with favourable and unfavourable quality of life (QoL)

	Total (*n* = 119)	Favourable QoL ODI < 12 (*n* = 35, 29%)	Unfavourable QoL ODI ≥ 12 *(n* = 84, 71%)	*p* value
	Median (IQR)	Median (IQR)	Median (IQR)	
Age (years)	67 (58;74)	65 (55;71)	68 (60;75)	0.530
Laboratory values				
CRP (mg/l)	46 (16; 132)	48 (10; 210)	46 (19; 120)	0.953
Leukocytes	8.8 (6.9; 11)	9.6 (7.5; 12.8)	8.5 (6.8; 10.3)	0.098
Creatinine (mg/dl)	0.8 (0.7; 1.1)	0.7 (0.6; 1.1)	0.87 (0.7; 1.1)	0.067
Haemoglobin (g/dl)	11.5 (10.2; 13.0)	12.0 (11.1; 13.4)	11.5 (10.2; 12.8)	0.644
Quick (%)	102 (87; 111)	103 (89; 112)	101 (87; 111)	0.767
Albumin (g/dl)	35.0 (29; 39)	35 (27; 40)	36 (30; 39)	0.456
Time to surgery (days)	4 (2; 9)	5 (2; 9)	4 (2; 9)	0.239
VAS back pain (T0)	8; (7;10)	8 (6;10)	8 (8;10)	0.889
VAS leg pain (T0)	6; (0;8)	3 (0;7)	7 (2;10)	0.014*
ODI (T0)	80; (64; 90)	73 (58; 89)	82 (67; 91)	0.363
COMI (T0)	9.3 (8.4; 10.0)	8.9 (7.7; 9.8)	9.5 (8.6; 10.0)	0.022*
	*n* (%)	*n* (%)	*n* (%)	
Gender				0.528
Male	78 (66)	23 (66)	54 (64)	
BMI				0.387
Underweight	7 (6)	4 (12)	3 (4)	
Normal	45 (39)	12 (35)	33 (40)	
Overweight	42 (36)	11 (32)	31 (38)	
Obese	22 (19)	7 (21)	15 (18)	
ASA				0.007*
< 2	49 (41)	21 (60)	28 (33)	
> 3	70 (59)	14 (40)	56 (67)	
Spinal level				0.524
Cervical	2 (2)	2 (2)	0 (0)	
Thoracic	35 (29)	11 (31)	24 (29)	
Lumbar	76 (64)	21 (60)	55 (66)	
Multilevel	6 (5)	3 (9)	3 (4)	
Segments affected				0.281
1	94 (79)	26 (74)	68 (81)	
> 1	25 (21)	9 (26)	16 (19)	
Bacteraemia	32 (27)	6 (17)	26 (31)	0.091
Pathogen detected	88 (74)	25 (71)	63 (75)	0.425
Pathogen				0.295
* S. aureus*	39 (44)	13 (52)	26 (41)	
CNS	21 (24)	3 (12)	18 (29)	
* Streptococcus* species	6 (7)	1 (4)	5 (8)	
GN	6 (7)	2 (8)	4 (6)	
* Proprionibacterium* species	4 (5)	1 (4)	3 (5)	
* Enterococcus* species	3 (3)	2 (8)	1 (2)	
Mycobacteria	3 (3)	2 (8)	1 (2)	
Anaerobes	2 (2)	0 (0)	2 (3)	
* Corynebacterium*	1 (1)	1 (4)	0 (0)	
Comorbidities/conditions				
Diabetes	18 (15)	5 (14)	13 (16)	0.557
Oncologic disease	19 (16)	6 (17)	13 (16)	0.509
Immunosuppression	7 (16)	4 (11)	3 (4)	0.112
COPD	10 (8)	1 (3)	9 (11)	0.117
IBD	2 (2)	0 (0)	2 (2)	0.497
Rheumatic disease	5 (4)	1 (3)	4 (5)	0.539
Cardiac insufficiency	12 (10)	2 (6)	10 (12)	0.254
Renal insufficiency	15 (13)	6 (17)	9 (11)	0.249
Alcohol abuse	8 (7)	3 (9)	5 (6)	0.434
Drug abuse	4 (3)	2 (6)	2 (2)	0.337
Neurological deficit	25 (21)	6 (17)	19 (23)	0.343
Frankel				
A	2 (2)	1 (3)	1 (1)	
B	4 (3)	1 (3)	3 (4)	
C	11 (9)	3 (9)	8 (10)	
D	8 (7)	1 (3)	7 (8)	
E	94 (79)	29 (83)	65 (77)	
Manifestations				
Endocarditis	1 (1)	0 (0)	1 (1)	0.706
Psoas abscess	26 (22)	27 (77)	66 (79)	0.521
Spinal empyema	50 (42)	16 (46)	34 (41)	0.372
Pretreatment				0.225
None	75 (63)	20 (57)	55 (66)	
Surgery	17 (14)	8 (23)	9 (11)	
Infiltration	27 (23)	7 (20)	20 (24)	
Surgical method				0.690
1 stage	50 (42)	16 (46)	35 (42)	
2 stage	68 (58)	19 (54)	49 (58)	

All statistical tests are two-tailed

*VA* visual analogue scale, *ODI* Oswestry Disability Index, *COPD* chronic obstructive pulmonary disease, IBD inflammatory bowel disease, *CNS* coagulase-negative staphylococci, *GN* Gram-negative bacteria, *ASA* American Society of Anesthesiologists score

^*^p value ≤ 0.05

One year after surgery (T1), 35 (29%) patients had favourable QoL (ODI < 12 points) and 84 (71%) patients had unfavourable QoL (ODI ≥ 12 points). Patients with favourable QoL more frequently had an ASA score ≤ 2 at baseline (60% vs. 33%; *p* = 0.007), a lower preoperative COMI score (8.9 vs 9.5; *p* = 0.022) and lower preoperative leg pain on the VAS scale (3 vs. 7; *p* = 0.014).

On multivariate analysis, younger age (hazard ratio = HR: 0.95; 95% CI 0.91–0.99; *p* = 0.022), lower albumin (HR: 0.9; 0.83–0.98; *p* = 0.019) an ASA score ≤ 2 (HR:4.24; 95% CI 1.42–12.68; *p* = 0.010), and a lower preoperative leg pain on the VAS (HR: 0.86; 95% CI 0.76–0.97; *p* = 0.018) were significantly predictive for favourable QoL after surgery (Table 2).

**Table 2:** Logistic regression model assessing the odds ratio for having a favourable outcome (ODI < 12) after surgical treatment after 1 year (t_1_)

	Odds ratio	95% CI for odds ratio		pp-value
		Lower bound	Upper bound	
Age	0.952	0.913	0.993	0.022*
Albumin	0.904	0.832	0.984	0.019*
Bacteraemia, yes (ref: no)	0.334	0.095	1.181	0.089
Immunosuppression, yes (ref: no)	0.178	0.026	1.242	0.082
Renal insufficiency, yes (ref: no)	0.283	0.073	1.102	0.069
VAS leg pain (t_0_)	0.858	0.756	0.974	0.018*
ASA ≤ 2 (ref: ≥ 3)	4.236	1.415	12.679	0.010*
Constant	2105.492			0.002

*VAS* visual analogue scale, *t*_*0*_ before surgical treatment, *ASA* American Society of Anesthesiologists score

^*^p value ≤ 0.05

## Discussion

In recent years, the incidence of VO increased due to improved diagnostics and demographic development showing an ageing population with chronic debilitating diseases [8–11]. VO frequently causes a profound impact on long-lasting back pain, function and QoL [12, 13]. The present prospective study showed a significant improvement of QoL in surgically treated VO patients after 1 year, but only about a third (29%) reached favourable QoL without restriction in daily life activities. Multivariable analysis revealed younger age, a lower ASA score, and less pre-operative leg pain as independent factors for favourable outcome.

Baseline characteristics in this study were typical and did not differ from other studies [8–10]. VO manifested predominantly in elderly men, back pain was present in almost all patients and the lumbar spine was mostly affected [8, 9, 14]. Also, the ODI showed a significant improvement after successful surgical treatment of VO after 1 year (T1), which is in line with the results from previous studies [1, 2, 15].

Previous studies have proved VO to be a severe disease with high mortality. The overall mortality has been reported up to 20% and appears to be particularly high in the first year after diagnosis [1, 11, 16]. A systematic review showed that neurological deficit and recurrence occur in one-third of cases after VO, respectively [17]. Available data on QoL after surgical treatment of VO are mainly based on retrospective analyses with small case numbers and reveal an improvement of QoL after surgery [2, 10, 15, 18]. However, after successful surgical treatment of VO, QoL levels remain below those of the normal population and functional outcomes as well as work ability are inferior compared with the normal population [10, 15, 16]. In this context, a systemic literature review on conservative and surgical treatment outcomes of VO proved in addition to high rates of orthopaedic and neurological complications a significant worsening in QoL [19].

In a previous study, we were able to confirm data from O´Daily, by proving that post-surgical QoL levels significantly improved, but only reached levels of QoL comparable to those from patients with chronic back pain [1]. This implies that statistically significant improvement in QoL scores is not necessarily equal to satisfactory clinical treatment success. For this purpose, not only relative changes, but clinically relevant thresholds in QoL scores should be considered in studies.

To date, the success of VO treatment is mainly based on functional or clinical factors, e.g. pain, neurological deficits, relapse and mortality [13, 18]. However, by focusing exclusively on clinical, functional or laboratory parameters, the generally poor outcome of VO is likely to be under-reported. It is important to note that in a complex clinical entity, such as VO, QoL may be reduced, even in the case of a supposedly successful therapy.

Various scores can be used to measure QoL in spinal disabilities, whereas the impact of spinal surgery is commonly evaluated with ODI, representing the internationally most recognized questionnaire for back pain. A comparison of the QoL scores Short-Form-Health 36, EuroQol-5D and ODI in degenerative disc disease patients pointed out that the ODI is the only disease- specific questionnaire and superior in the ability to diagnose clinical improvement [20].

Against the background of a lack of a generally valid cutoff value definition of therapeutic success or favourable QoL after VO therapy, we have adapted the cutoff values from chronic back pain without infection for VO. In 2000, Fairbank et al. were the first group, who established a normative ODI score for patients with acute and chronic back pain [3]. Tonosu et al. estimated cutoff values for the ODI for patients with back pain. They stated that an ODI score between 0 and 12 is associated without disability, allowing a normal daily life participation, whereas a score ≥ 12 represents a condition of back pain with disability that leads to restrictions in daily life [4]. Based on this cutoff value, we defined a favourable ODI for VO patients to be 12. The follow-up period was set to 1 year post-surgery, since we were able to prove in a previous study that QoL scores reached a plateau within 1 year after surgery and do not change significantly afterwards [1].

Several retrospective evaluations show high age and a high ASA score are associated with a poor outcome in VO [21–24]. These findings were in line with our results, as lower age and lower ASA score were associated with favourable QoL. In contrast, a pre-operative neurological deficit had no significant influence on favourable QoL. In both subgroups, a severe preoperative neurological deficit (Frankel A-C) was present in 15% of the VO patients, which is consistent with the findings in the previous literature [10, 25, 26]. Transferring these data to a clinical context, VO patients can achieve a favourable QoL by means of ODI despite a severe preoperative neurological deficit.

Surprisingly, a low albumin value is supposed to be a predictive value for favourable QoL. However, the clinical sense of this observation is questionable, since a low albumin value is primarily an indicator for cachexia. Looking at the median values of the two subgroups (35 vs 36), it is clear that the difference of one point is not clinically significant and therefore does not require further attention.

Interestingly, a lower preoperative leg pain measured by VAS was also associated with favourable QoL. Recent studies have already proved that back pain plays an important role as the key symptom of VO. Our results pointed out that leg pain is not only important, but also an independent predictive factor for favourable QoL. In 2019, Kim et al. already showed the importance of radiating pain distinguishing postoperative VO (PVO) from native VO (NVO) significantly, with NVO as the more severe disease with a higher mortality [27].

The strengths of the current study are the prospective study design with uniform 1-year follow-up that yields more detailed and robust information with strict inclusion criteria compared to case series or retrospective cohort studies. By focusing on the threshold of the ODI to define favourable QoL, our findings add important new clinical relevant information.

This study's limitations include possible selection bias, since it is a single-centre study from a tertiary care hospital primarily treating multimorbid patients and/or patients with complicated case histories. By excluding patients having died within 1 year after surgery, other predictive factors might be missed. We had to transfer threshold values of ODI from acute and chronic back pain to VO, as specific values are missing; nevertheless, a transfer is reasonable as it is known that even after successful treatment the QoL of VO patients is comparable to that of those suffering from chronic back pain. In the future, specific thresholds for QoL scores in VO patients need to be defined.

## Conclusion

One-third of surgically treated VO patients (29%) in our cohort achieved favourable QoL by means of ODI. In addition to the known factors, age and ASA score, we were able to demonstrate that even preoperative leg pain was able to predict favourable outcome, so that greater attention should be paid to this parameter. On the other hand, preoperative neurologic deficit was not predictive of QoL after surgical treatment of VO. Our findings can already facilitate an estimation of the prognosis when informing the patient before surgery, and underscore that spine disability questionnaires, such as ODI, measuring QoL are mandatory to evaluate comprehensively the outcome of this entity.

## Data Availability

(Software application or custom code). Not applicable.

## Availability of data and material

The datasets used and/or analysed during the current study are available from the corresponding author on reasonable request.
